# The prevalence of terraced treescapes in analyses of phylogenetic data sets

**DOI:** 10.1186/s12862-018-1162-9

**Published:** 2018-04-04

**Authors:** Barbara H. Dobrin, Derrick J. Zwickl, Michael J. Sanderson

**Affiliations:** Department of Ecology and Evolutionary Biology, University of Arizona, 1041 E. Lowell St, Tucson, AZ 85721 USA

**Keywords:** Terrace, Partitioned model, Data decisiveness, Phylogenetics, Supermatrix, Large phylogenetic trees

## Abstract

**Background:**

The pattern of data availability in a phylogenetic data set may lead to the formation of terraces, collections of equally optimal trees. Terraces can arise in tree space if trees are scored with parsimony or with partitioned, edge-unlinked maximum likelihood. Theory predicts that terraces can be large, but their prevalence in contemporary data sets has never been surveyed. We selected 26 data sets and phylogenetic trees reported in recent literature and investigated the terraces to which the trees would belong, under a common set of inference assumptions. We examined terrace size as a function of the sampling properties of the data sets, including taxon coverage density (the proportion of taxon-by-gene positions with any data present) and a measure of gene sampling “sufficiency”. We evaluated each data set in relation to the theoretical minimum gene sampling depth needed to reduce terrace size to a single tree, and explored the impact of the terraces found in replicate trees in bootstrap methods.

**Results:**

Terraces were identified in nearly all data sets with taxon coverage densities < 0.90. They were not found, however, in high-coverage-density (i.e., ≥ 0.94) transcriptomic and genomic data sets. The terraces could be very large, and size varied inversely with taxon coverage density and with gene sampling sufficiency. Few data sets achieved a theoretical minimum gene sampling depth needed to reduce terrace size to a single tree. Terraces found during bootstrap resampling reduced overall support.

**Conclusions:**

If certain inference assumptions apply, trees estimated from empirical data sets often belong to large terraces of equally optimal trees. Terrace size correlates to data set sampling properties. Data sets seldom include enough genes to reduce terrace size to one tree. When bootstrap replicate trees lie on a terrace, statistical support for phylogenetic hypotheses may be reduced. Although some of the published analyses surveyed were conducted with edge-linked inference models (which do not induce terraces), unlinked models have been used and advocated. The present study describes the potential impact of that inference assumption on phylogenetic inference in the context of the kinds of multigene data sets now widely assembled for large-scale tree construction.

## Background

Among the methodological challenges in phylogenetic inference are those posed by missing data. Problems tied to incomplete data sets first emerged in the context of paleontological data matrices [1–3], from which character states may be missing because of inapplicable characters or fossil incompleteness, leading to parsimony reconstruction (used widely for morphological data sets) recovering multiple, equally good trees. A large literature (e.g., [4–16]) has since assessed the risks and identified advantages linked to the use of incomplete data sets for inference, and the issues remain salient in the modern phylogenetics context because few data sets are fully sampled (i.e., include data at every taxon-by-gene position). Incomplete data can be analyzed accurately [10, 12, 14, 16–18] but studies also find that sparse data can undermine phylogenetic accuracy [4–6, 8] and confidence [9, 19, 20]. Recent work shows, for example, that abundant or nonrandom missing data can bias estimates of model parameters [21] promote the emergence of support artifacts [22, 23]; and worsen biases built into heuristic search procedures [24, 25], leading to artifactual tree search outcomes [25].

Adding to these difficulties are terraces [26, 27], collections of equally optimal trees that may arise in tree space because of the taxon coverage patterns (the pattern of gene presence/absence across taxa) in partitioned alignments, such as commonly are found in multigene data matrices. Terraces can slow tree search [26, 28] and mislead heuristic search algorithm [27]; when a tree search algorithm returns one putatively optimal tree that is actually on a terrace, then this adds ambiguity to tree inference. The presence of terraces can also confound confidence assessment: in bootstrapping (under some conditions), replicates are more likely to return a spurious clade if the clade occurs frequently on a terrace of optimal trees; and in Bayesian assessment, long-branch bias in the presence of missing data can elevate posterior probabilities of some of the trees belonging to a terrace [27]. The latter “phantom” support phenomenon resembles the “star paradox” [29] and Bayesian long-branch repulsion effects [30] observed elsewhere.

**Fig. 1:**
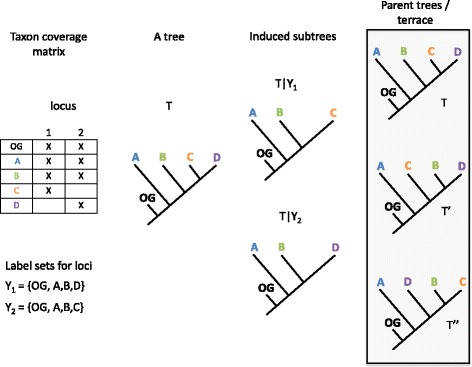
Origin of terraces. The two-locus taxon coverage pattern (displayed as a taxon coverage matrix, left) of a data alignment induces two subtrees, *T|Y*_1_ and *T|Y*_2_, of a tree, *T*, inferred from the data. Three trees, *T, T’,* and *T”* all display and are parent trees of *T|Y*_1_ and *T|Y*_2._ If the tree scoring function is parsimony or ML-EUL, the scores of *T, T’,* and *T”* are identical, and the trees constitute a terrace. Figure adapted from [27]. Credit: J. Charboneau

Precise necessary and sufficient conditions for the occurrence of terraces have been described elsewhere [26, 27]. Roughly speaking they include: 1) the tree optimality criterion is parsimony or partitioned maximum likelihood (ML) and, if the latter, edge lengths are optimized independently across data partitions (i.e., the inference model is “edge-unlinked” (EUL)) [27]; and 2) each partition is sampled for fewer than the full complement of taxa. For any “parent” tree, *T*, having all the taxa in a data matrix, each partition of the matrix with fewer than this number of taxa sampled induces (“displays”) a subtree of *T* with those “missing” taxa pruned. Depending on the taxon coverage pattern, these subtrees may be compatible not only with *T* but with an assortment of other parent trees, each displaying the induced subtree (Fig. 1). If the optimality function is one of those cited above, scores of all parent trees will be identical [26, 27], and collectively the parent trees are called a terrace. Because terraces consist of parent trees that display the same compatible subtrees, they can be characterized using algorithms from the supertree literature; in particular, terraces can be discovered and described without the need to search tree space once the first tree, *T*, is found [26, 27].

All else being equal, terraces should arise more often from data sets with sparser taxon coverage, and more often when data span many taxa and few genes (as in a “tall” matrix), than the converse (as in a “wide” matrix) [31]. The increased prevalence of next-generation sequencing (NGS) sampling approaches will reduce the incompleteness of data matrices, but “gappiness” currently characterizes much large-scale phylogenetic data, for reasons including 1) the use of public sequence archives, which store disparate data sets composed of different taxa and different numbers of taxa; 2) biological [30] or methodological [32] barriers to obtaining orthologous sequences; 3) the use of shallow coverage protocols with NGS methods; and 4) loss of genes. In this paper, we investigate the terraces that would arise from 26 large data sets under the necessary inference assumptions. In particular, we investigated whether the published optimal trees – generally maximum likelihood trees - were on a terrace, and the properties of those terraces. When we reviewed the methods and models used originally to recover the trees, we found surprising variability: one author [33] conducted unpartitioned analysis, another [34] reported having used an edge-linked (EL) model, and many authors left inference model details unspecified. Of those in the latter category, some may have used linked edge-length parameters (and consequently EL models), often the default parameter setting of tree reconstruction programs. However, we were less interested in evaluating the findings of the published studies than in constructing a test bed of data sets for examining the size and diversity of terraces that would emerge under EUL inference (or parsimony). EUL models have been used in likelihood-based tree reconstruction, and may confer advantages in analysis of some time-heterogeneous data sets (see Discussion); and terraces are predicted to emerge from incompletely-sampled data under EUL assumptions. Accordingly, we evaluated the terraces that would have arisen if the reported trees had been products of EUL maximum likelihood inference (ML-EUL) or parsimony. We characterized the terraces, measuring their size and the diversity of their trees. We examined terrace properties in relation to the data availability characteristics of the data sets, including taxon coverage density and a measure of data sampling sufficiency derived from theory. When bootstrapping to obtain tree support values, each replicate tree may belong to a terrace. We used consensus methods to measure the impact of these terraces on bootstrap support. Finally, we examined terrace size as a function of a simple measure of overall data coverage, the percentage of taxon triples sampled within partitions. Because terrace formation in likelihood inference occurs with the use of EUL models, we also used the Akaike Information Criterion (AIC) [35] to identify the more suitable model (EL or EUL) for each data set in the study sample.

## Methods

### Concepts and definitions

Earlier articles [26, 27] provide detailed exposition of terraces and their properties. Here, we outline terrace theory in brief.

#### Terraces and inference models

Consider a data matrix consisting of aligned, homologous sites (these may be nucleotides or other characters) and *n* taxa, with the sites subdivided into *k* loci. We may denote the set of *n* taxon labels as *X*. Each locus corresponds to a unit such as a gene or a codon position, or perhaps to a collection of sites demarcated by some a posteriori criterion. Throughout this article, we will refer to loci variously as “loci,” “partitions,” and “genes,” without regard to the scheme used to cluster the data. If *any* data are present for a taxon at a locus, we consider that locus sampled for the taxon. The *coverage pattern S* for the data and partitioning scheme consists of the subsets of taxa *Y*_1_,...,*Y*_k_ sampled for each of the *k* loci. *Taxon coverage density*, or just *coverage density*, refers to the percentage of taxon-by-locus combinations that have any data present. We also speak of a *taxon coverage matrix*, which differs from the coverage pattern only in that it records the presence and absence of samples at taxon-by-locus locations.

Given a tree *T* on *X*, each of the taxon subsets *Y*_j_ in *S* induces a subtree *T|Y*_j_ composed only of taxon labels in *Y*_j_ - that is, *T|Y*_j_ is the subtree of *T* remaining after all taxa not present in *Y*j are removed. The tree T displays the set of induced subtrees *T|Y*_1_,…,*T|Y*_k_, and is a parent tree of *T|Y*_1_,…,*T|Y*_k_. Fig. 1 illustrates how more than one tree may display (i.e., be a parent tree of) a set of subtrees induced by a taxon coverage pattern: the two-locus coverage pattern *Y*_1_, *Y*_2_ induces the subtrees *T|Y*_1_, *T|Y*_2_, of which *T, T’,* and *T”* are parent trees. If the parent trees are scored with an optimality function such as parsimony or maximum likelihood, and if all parent trees score the same, collectively the parent trees are called a terrace.

If the scoring criterion is parsimony, the set of parent trees is always a terrace [26]. If the criterion is maximum likelihood (ML), the parent trees are a terrace if edge-length parameters of the inference model are optimized independently across loci [27]. In this paper, we refer to models with such parameters as edge-unlinked (EUL). An edge-linked (EL) model has a single length parameter per edge (i.e., optimization is joint across loci). A partially edge-linked (PEL) model joins edge-length parameters across loci by one or more proportionality constants. Use of an EUL model is a sufficient condition for the emergence of terraces, while optimization with a linked model (EL or PEL) is insufficient – terraces do not arise under their assumptions. No conditions apply to the rate matrix of the model, which may be defined independently or jointly across loci [27].

As noted in the Introduction, often we could not discern rigorously the details of the inference models used in the phylogenetic studies in our sample. In particular, authors often left unspecified the linkage type (i.e., whether optimized jointly [linked] or separately [unlinked] across partitions) assigned to edge-length parameters, and some authors may have relied on inference tool default settings. In RAxML [36], the program used most often across the sample, parameters for edge length are linked by default, implementing a model (EL) that does not induce terraces. The authors of one analysis [34] explicitly reported having used an EL model. As we have noted, we were more interested in the impact of the structure of the data than the particular inference assumptions of the published papers, and accordingly, we investigated the terraces that would have arisen had the reported trees been recovered with parsimony or with some form of ML-EUL inference model.

#### Defining and decisiveness

If a tree *T* on *X* is the only parent tree of a set of subtrees induced by a coverage pattern *S*, we say that the subtrees *define T*. Similarly, a coverage pattern *S* is said to be *decisive* for *T* if *T* is the only parent tree of the subtrees induced by *S*. Theory [31, 37] establishes necessary and sufficient conditions under which a coverage pattern achieves decisiveness. A theory of defining sets out conditions under which a set of subtrees define a tree. Here we summarize a selection of these theoretical results, described previously in [31]:

- For a coverage pattern *S* to be decisive for all (unrooted) trees on *X*, it is *sufficient* that one locus is fully sampled (i.e., for every label in *X*). This condition follows trivially from a condition (which we do not describe here) applying to the distribution of taxon quadruples among label subsets in *S*.
- For a coverage pattern *S* to be decisive for all (unrooted) trees on *X*, it is *necessary* that every triple of taxa (set of 3 taxa) is present (i.e., sampled or observed) in at least one of the taxon subsets in *S*.

The latter result suggests intuitively that the distribution of triples in a coverage pattern, and the number of parent trees that can be constructed from its induced subtrees, may be empirically correlated. Sanderson et al. 2010 [31] speculated that the percentage of observed taxon triples might indeed predict the impact of a given quantity of missing data. Further theory developed in [31, 38] similarly suggests such a relationship. We state here one such further result, given in [38]:

- Given a rooted tree *T* and a coverage pattern *S*, the set of induced subtrees *T|Y*_1_,…,*T|Y*_k_ defines *T* if every edge of *T* is *distinguished* by some rooted triplet from *T|Y*_1_,…,*T|Y*_k_. To describe the concept of *distinguishing* informally, let *π* be a leaf taxon whose incident edge subtends the root of *T*, but which is not found in *X* (i.e., the label set of *T)*; let *a*, *b*, and *c* be taxa belonging to *X.* The rooted triplet *a|bc* distinguishes an edge *e* of *T* if each taxon in the set {*π,a,b,c*} has one label found in each subtree in *T* whose roots are adjacent to *e*, and *e* corresponds to the edge of the resolved quartet *πa|bc.*

Whether a taxon triple is associated with a distinguishing triplet depends on the shape of *T*, but taxon triple percentage can be thought of as a (numerically smaller) proxy for the proportion of edges of *T* distinguished by rooted triplets. Edges not fixed by induced subtrees can be broken and their subtended partial trees placed elsewhere, forming equally optimal alternative topologies.

### Terrace discovery and analysis

#### Selection and preparation of empirical data sets

From recent phylogenetics literature, we selected 13 multi-locus data sets, each consisting of at least 7 loci and at least 95 taxa [33, 34, 39–48]. From the largest of these, the ~ 33,000-taxa vascular plant “megamatrix” of Zanne et al. [42], we extracted 13 disjoint data subsets, each corresponding to a named genus or family, and each including (with one exception) at least 95 taxa. Some of these data subsets contained fewer than the 7 loci present in the megamatrix. Across all data sets (including vascular plant subsets), the number of taxa ranged from 57 to 7000, the number of loci from 5 to 1122, and the number of aligned sites from 5054 to 504,850. Taxon coverage densities ranged from 0.06 to 0.98 (Table 1). Of the studies selected, all but two reported maximum likelihood trees. We explored the terraces (if present) associated with these trees, characterizing terraces as they would have arisen had the published trees been products of parsimony or ML-EUL. To analyze the data set of [44], we used the published maximum clade credibility (MCC) Bayesian tree. To analyze the data set of [34], we used the published partitioning scheme and a tree that we constructed ourselves from the aligned data using parsimony heuristic search in PAUP [49]. [34] reported a tree estimated from the data (with an edge-linked (EL) model), but no machine-readable copy of the tree accompanied the article. For each data subsample of the plant megamatrix, we extracted the corresponding subtree from the ~ 33,000-taxa megaphylogeny. Polytomies were absent from all trees except that reported by [33].

**Table 1:** Data set profiles and results of terrace and decisiveness analyses

Taxon	Number of taxa	Number of loci	Number of sites^	Taxon coverage density	Terrace size	*ρ* (resolution) of strict consensus of trees on terrace	Min. loci needed for decisiveness (*kmin), p* = .05	Gene (locus) sampling sufficiency (*ζ*)	Ref.
Birds	7000	32	39,611	0.12	1.30E + 388	**	129,035	−8.3	[39]
Lichenized fungi	1317	9	7433	0.44	11,655	**	574	−4.16	[40]
Saxifragales	946	51	48,242	0.06	**	NA	2,107,107	−10.63	[33]
Bats	815	29	20,364	0.15	1.43E + 41	0.78	44,209	−7.33	[41]
Rosaceae*	529	7	11,728	0.3	1.72E + 23	0.77	2627	− 5.93	[42]
Primates	372	79	61,198	0.37	70.8 million	0.92	982	− 2.52	[43]
Caryophyllaceae*	225	7	11,753	0.29	718.3 billion	0.77	2349	− 5.82	[42]
Scincid lizards	213	6	5283	0.78	3	~ 1.00	37	−1.83	[44]
Chameleons	202	6	5054	0.92	1	1	14	−0.83	[45]
Solanum*	187	7	11,875	0.31	211.9 million	0.68	1730	− 5.51	[42]
Primula*	185	6	9408	0.43	2835	0.92	466	−4.35	[42]
Ranunculus*	170	7	9504	0.31	3	0.99	1889	− 5.6	[42]
Mammals	169	26	35,600	0.94	1	1	11	0.86	[46]
Insects	144	479	413,459	0.95	1	1	10	3.88	[34]
Iris*	137	6	8098	0.33	1	1	1384	−5.44	[42]
Eucalyptus*	136	6	7512	0.23	27	0.9	5416	− 6.81	[42]
Asplenium*	133	6	9797	0.21	95	0.64	8269	−7.23	[42]
Euphorbia*	131	7	11,648	0.28	759	0.87	2681	−5.95	[42]
Rhododendron*	117	7	9536	0.35	81	0.95	1034	−5	[42]
Ficus*	112	5	5645	0.36	851,445	0.8	12,357	−7.81	[42]
Syzygium*	106	5	5775	0.35	45	0.96	994	−5.29	[42]
1000 Plants.1	102	8	290,719	0.97	1	1	7	0.15	[47]
1000 Plants.2	102	620	290,719	0.91	1	1	13	3.88	[47]
Caryophyllales.1	95	209	87,082	0.98	1	0.99	8	3.23	[48]
Caryophyllales.2	95	1122	504,850	0.92	1	0.99	12	4.56	[48]
Allium*	57	6	6938	0.24	973,215	0.32	4231	− 6.56	[42]

^: Site counts do not include alignment columns containing no data, and may differ from counts reported in the original literature. *k*_*min*_: theoretical minimum number of loci that would need to be sampled to guarantee that a data set of a given sampling density and taxon count would be decisive for a random, unrooted tree, assuming random distribution of taxon samples. *ζ*: gene (locus) sampling depth “sufficiency”: *k*_min_ normalized to the number of genes (loci) sampled and transformed for scale. *ζ* < 0 indicates insufficient sampling depth, *ζ* > = 0 sufficient sampling depth. * denotes a subsample of the Zanne et al. [42] vascular plant “megamatrix”. **: tree enumeration or consensus tree construction terminated prior to program completion

Several of the published data alignments included sequences for taxa not found in the accompanying trees. We deleted these taxa; consequently, some taxon counts in our experimental data sets differ from the published counts. We also deleted a small number of additional taxa (three or fewer across all data sets) when we encountered difficulties processing their sequence data into the format required for terrace analysis. All final data alignments, partitioning schemes, and trees analyzed for this study have been posted on the GitHub website.

#### Discovering and characterizing terraces

We used the Python program ‘terraphy’ [50], written by DJZ, to discover and characterize the terraces. Terraphy accepts as input a data matrix of aligned sites, a partition scheme, and a tree. It computes the taxon coverage matrix for the alignment and partitioning scheme, the size of the terrace to which the tree belongs, and the strict and Adams (BUILD) [51] consensus trees of the trees on the terrace. To compute terrace size, terraphy uses the Constantinescu & Sankoff [52] supertree algorithm, created to construct the full set of parent trees of a group of compatible input trees. To compute the Adams consensus tree, the program uses the BUILD algorithm of Aho [51]. To construct the strict consensus tree, the program relies on algorithms of Constantinescu & Sankoff [52] and Steel [38]. The scaling properties of these operations have been described in [26, 27]. The terraphy package also includes functionality to: 1) construct and output samples of trees from a given terrace, 2) determine whether two trees (found in bootstrap replicates, for example) belong to the same terrace, and 3) report the number of equally good subtree resolutions within each clade in the strict consensus tree of a terrace.

Terraphy treats input tree polytomies as “soft” or irresolvable. When the program receives a nonbinary tree, it evaluates the terraces of the alternative polytomy resolutions, and its output is the sum of tree counts from those terraces. The impact of polytomies on terrace tree counts is minimally relevant to this study because all but one of the trees we examined were binary.

#### Variability among trees on terraces

To describe the diversity of trees on the terraces, we constructed the strict consensus tree of each terrace and calculated its resolution, *ρ* (defined as the ratio of the number of a tree’s bipartitions to the number of bipartitions of a fully resolved (binary) tree of the same size).

#### Number of loci that must be sampled to ensure decisiveness; “gene sampling sufficiency”

A probability model of random taxon sampling, described in [31, 53], predicts the lower bound on the number of loci, *k*_min_, that would need to be sampled to guarantee that a taxon coverage pattern, *S*, given its taxon coverage density and taxon number, *n*, would be decisive for some (random) tree constructed on the label set, *X,* of *S*:

$$ {k}_{\mathrm{min}}=\frac{\ \ln \left(\left(\begin{array}{c}n\\ {}3\end{array}\right)/p\right)\ }{-\ln \left(1-{d}^4\right)} $$

which approximates to

$$ {k}_{\mathrm{min}}\approx \frac{\log \frac{n^3}{6p}}{-\log \left(1-{d}^4\right)} $$

([53], Mike Steel, personal communication, 2015), where *d* is the taxon coverage density of *S*, *n* the number of taxa in *S*, and *p* the desired confidence level. Henceforth, *k*_min_ stands for the approximation. To compare data sets, we used a normalized value that we call “gene sampling sufficiency” (i.e., the depth of the gene sampling), or *ζ*:

$$ \zeta =\ln \kern0.28em \frac{k_{\mathrm{min}}}{k} $$

where *k* is the number of loci (partitions) sampled. If decisiveness for a random tree on *X* is highly probable (*p* < 0.05), then *ζ* ≥ 0. Otherwise, *ζ* < 0.

#### Impact of terraces on bootstrap support

**Fig. 2:**
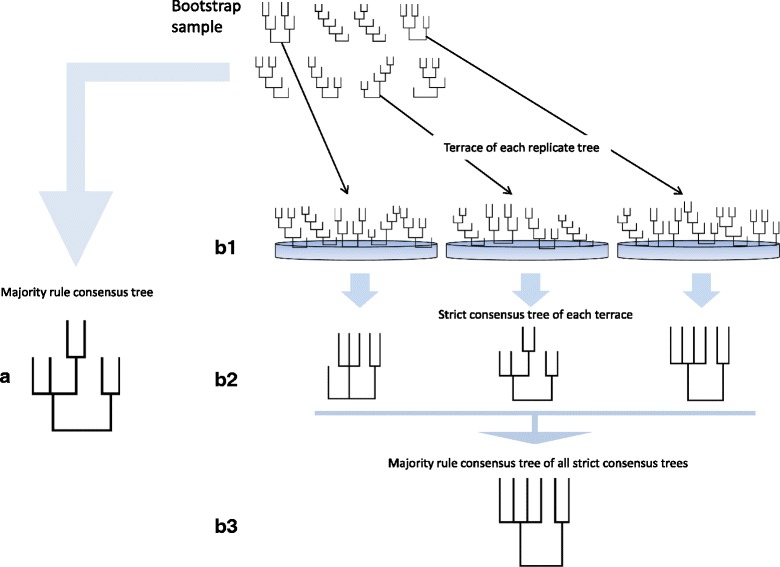
Measuring “terrace-aware” bootstrap support. Top center of figure: a sample of bootstrap replicate trees. **a**. Majority rule consensus tree of replicate set. **b1**. Terraces containing the individual replicate trees. **b2**. Strict consensus tree of each terrace. **b3**. Majority rule consensus tree of the strict consensus trees, or “terrace-aware” majority rule tree

In bootstrapping, the tree returned by each bootstrap replicate may be part of a terrace of equally good trees. To examine the impact of terraces on resampling support, we selected three small-to-medium-sized (112–225 taxa) data sets whose terraces were among the larger of those recovered. From each of the three data sets, we constructed 100 RAxML rapid bootstrap trees. We used PAUP to construct a majority rule consensus tree of each bootstrap replicate set and computed *ρ* for each majority rule consensus tree (Fig. 2). Next, using terraphy, we evaluated the terrace of each replicate tree and constructed the strict consensus tree of each terrace. Finally, we used PAUP to construct the majority rule consensus tree of each collection of strict consensus trees. We call these majority rule trees “terrace-aware,” because they exclude clades present in fewer than 100% of trees on the terrace found in each bootstrap replicate. We computed *ρ* for each “terrace-aware” consensus tree.

#### Observed taxon triples and terrace size

To test the conjecture of Sanderson et al. [31] that the fraction of triples sampled in taxon subsets (i.e., *Y*_1_,*...,Y*_k_) might predict the effects of a given amount of missing data, we computed the observed triple proportion (see Concepts and Definitions, earlier) for 12 data sets of relatively similar taxon coverage density and *ζ* values. Taxon coverage densities for this group ranged from 0.19 to 0.43, and *ζ* values ranged from − 4.35 to − 7.81. All data sets were chosen from among the vascular plant subsamples.

#### Edge-length model choice

Although terraces are only known to occur with EUL models, EL may not be the best model for all data sets. We used the Akaike Information Criterion (AIC) [35] to identify the most appropriate edge-length model for each data matrix in the study sample. For each matrix, we obtained maximum likelihood scores for a tree previously inferred from the data (in each case the tree used for terrace analysis) with both models. For likelihood analyses, we used RAxML v. 8.2.11 [36], with separate HKY85 [54] substitution matrices for each partiton of DNA data sets, and separate WAG [55] transition models for each partition of protein data sets. We used the GAMMA model of rate heterogeneity for all data sets. Within pairs of inference models, the EUL model differed from the corresponding EL model only in estimating branch lengths independently across loci. We computed ΔAIC [56, 57] for each pair of models.

## Results

### Size of terraces; relationship to taxon coverage percentage

**Fig. 3:**
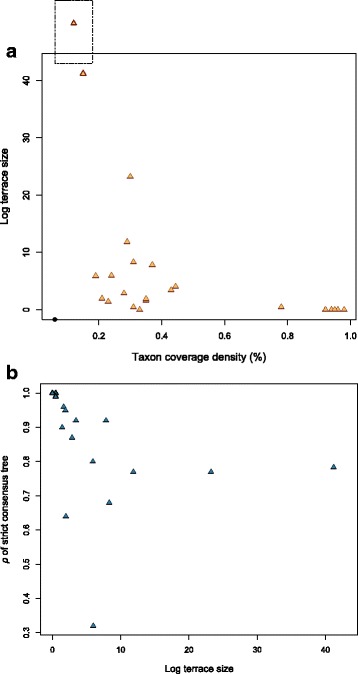
Terrace properties. **a**: Terrace size as a function of taxon coverage density. The black dot marks the coverage density of the data set for which we terminated terrace tree enumeration before completion (see text). The data point above the plot (in dashed box) represents the largest terrace measured, 10^388^ trees. **b**: Resolution (*ρ*) of the strict consensus tree of a terrace plotted as a function of terrace size. *ρ* is defined as the ratio of the number of a tree’s bipartitions to the number found in a binary tree of the same size. For two terraces identified in data sets, including the largest (10^388^ trees) and a terrace consisting of 11,655 trees, we terminated construction of the strict consensus tree before completion

We succeeded in measuring the terraces present in 25 data sets; the sizes ranged from one tree (a nominal terrace) to an astonishing 10^388^ trees (Fig. 3a, Table 1). The latter terrace was that found in the 7000-taxon data matrix, the largest (in terms of taxa) of those analyzed. In evaluating the terrace of the large (946 taxa), low-sampling-density (coverage density of .06) data set of [31], we terminated the program run after several weeks. Although the time required to count trees on this terrace implies that it is very large (as run time scales linearly with terrace size [26, 27]), the polytomy topology (*ρ* = 0.82) of the tree may have extended the program running time (see Methods). No terrace of a data set of coverage density greater than 0.90 exceeded one tree. For the 13 plant “megamatrix” data subsets, terraces ranged in size from 1 to ~ 10^23^ trees, although taxon coverage densities for these data sets spanned a narrow range, from 0.19 to 0.43. In general, terrace size varied inversely with the taxon coverage density of the data (Fig. 3a).

### Minimum gene sampling depth needed for decisiveness

**Fig. 4:**
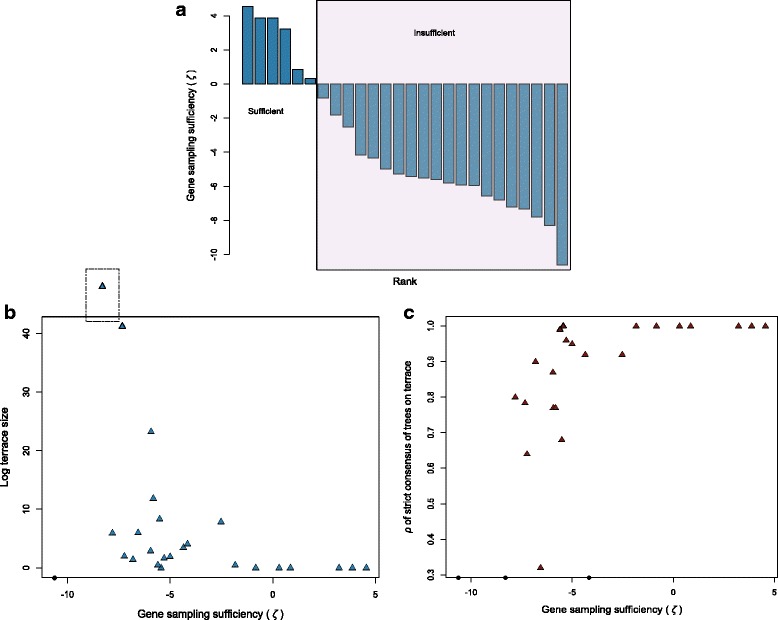
Gene sampling sufficiency (*ζ*) and terrace properties. *ζ*: scaled ratio of the number of genes present in the data set and the theoretical minimum number of genes needed for the data set to be decisive. A decisive data set induces a terrace of only one tree. *ζ* > =0: number of genes is sufficient for the data to achieve decisiveness. *ζ* < 0: number of genes is insufficient. **a**. Rank-order *ζ* of the 26 data sets. The number of genes is sufficient to achieve data decisiveness in only 6 data sets. **b**. and **c**. Terrace size and consensus tree *ρ* plotted as a function of *ζ*. The data point above the plot in **b**. represents the largest, 10^388^-tree terrace. Black dots mark the *ζ* values of data sets for which we terminated terrace tree enumeration or consensus tree assembly before completion

*k*_min_ was often very large, exceeding 1000 loci for 16 data sets, and exceeding 2 million loci for one data set (Table 1). Sampling sufficiency, *ζ,* measured less than zero (i.e., insufficient) for all but six data sets (Fig. 4a, Table 1). As with taxon coverage density, terrace size generally varied inversely with sampling sufficiency (*ζ*) (Fig. 4b). Terraces found in two data sets for which values of *ζ* were low (*−* 5.44 and − 5.60) comprised 1 and 3 trees, respectively, results at odds with the predictions of the Steel [53] and Sanderson et al. [31] probability model. The uniform taxon sampling assumed by their model, however, may not reflect samples found in many empirical data sets.

### Variability among trees on terraces

After several weeks we terminated construction of the strict consensus tree of both the 11,655-tree terrace associated with the data set of [40], and the enormous 10^388^ tree terrace found in the data set of [39]. Although time to compute the strict consensus tree of a terrace scales polynomially**,** the large sizes of the tree (1337 taxa) on the one hand, and the terrace on the other, likely explain the long run times required to complete the program runs. For the remaining terraces, except those of one tree (nominal terraces) and the terrace discovered from the smallest data set, *ρ* of the strict consensus trees ranged from 0.64 to 0.98 (Fig. 4c, Table 1). In general, *ρ* varied inversely with terrace size (Fig. 4b); exceptions included the value *ρ* = 0.92 for the ~ 71 million-tree terrace associated with the data set of [43]. *ρ* measured less than 0.50 only for the consensus tree associated with the smallest data set, a 57-taxon subsample of the Zanne et al. 2014 [42] matrix.

### Impact of terraces on bootstrap support

**Fig. 5:**
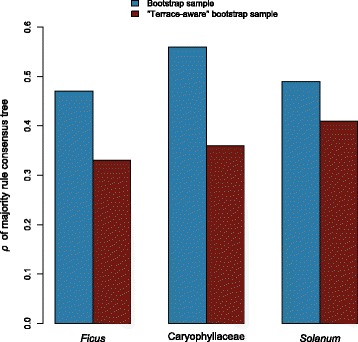
Comparing bootstrap and “terrace-aware” bootstrap support. A “terrace-aware” bootstrap sample consists of 100 strict consensus trees, each the consensus of the terrace associated with an individual bootstrap replicate tree. The ordinary bootstrap sample consists of 100 replicate trees. *ρ* was computed for the majority rule consensus trees of the two samples

The resolution, *ρ*, of the bootstrap majority rule consensus trees measured 0.47, 0.49, and 0.56, while *ρ* for the “terrace-aware” majority-rule trees measured 0.33, 0.41, and 0.36, respectively (Fig. 5).

### Percentage of taxon triples observed and terrace size

**Fig. 6:**
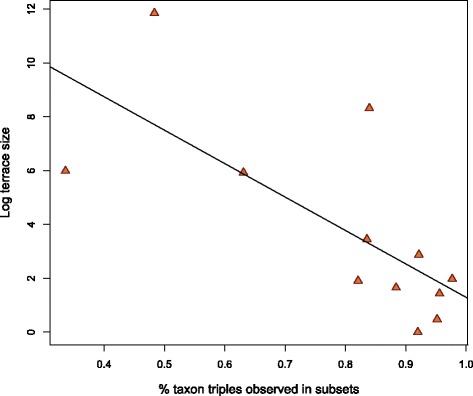
Terrace size and distribution of taxon samples. Terrace size plotted against the fraction of taxon triples observed in locus subsets (see text) for 12 data sets of similar taxon coverage densities and *ζ*. Linear regression line *p* = .008. Range of taxon coverage density values: 0.19–0.43. Range of *ζ* values: − 4.35 – − 7.81 (*ζ*: gene sampling sufficiency [see text])

As anticipated [31], the percentage of triples sampled within taxon subsets (*Y*_1_,…,*Y*_k_) varied inversely (*p* = .008) with terrace size among data sets of similar sampling density and *ζ* (Fig. 6).

### Edge-length model choice

The AIC criterion favored the EUL model over the corresponding EL model for 9 data sets (Table 2), including phylogenomic and high-coverage-density data matrices from which terrace analysis recovered terraces of one tree. The EL model was preferred for the remaining data sets, including all but one of the 13 vascular plant subsets.

**Table 2:** EL and EUL model choice

Taxon	Number of taxa	Taxon coverage density	Number of loci	Model	Number of parameters	Log likelihood	AIC	AIC [EL] – AIC [EUL]#	Terrace size
Insects	144	0.95	472^	EL	757	−30,914,676	61,830,867	417,292	1
				EUL	134,992	−30,571,795	61,413,574		
Caryophyllales.2	95	0.92	1120^^	EL	1307	−20,001,090	40,004,795	184,811	1
				EUL	210,560	−19,699,432	39,819,984		
1000 Plants.2	102	0.91	620	EL	5781	−8,873,326	17,758,215	136,442	1
				EUL	130,200	−8,680,687	17,621,773		
1000 Plants.1	102	0.97	8	EL	273	−8,881,093	17,762,731	43,552	1
				EUL	1680	−8,857,910	17,719,179		
Caryophyllales.1	95	0.98	209	EL	396	−3,490,762	6,982,316	30,510	1
				EUL	39,292	−3,436,611	6,951,806		
Mammals	169	0.94	26	EL	569	−1,227,939	2,457,016	18,145	1
				EUL	8944	−1,210,492	2,438,871		
Chameleons	202	0.92	6	EL	455	− 188,780	378,471	4565	1
				EUL	2460	−184,493	373,906		
Scincid lizards	213	0.78	6	EL	477	−129,669	260,291	1410	3
				EUL	2592	− 126,849	258,881		
Euphorbia*	131	0.28	7	EL	322	−46,304	93,252	435	759
				EUL	1876	−44,533	92,817		
Iris*	137	0.33	6	EL	311	−29,602	59,827	−46	1
				EUL	1596	−28,340	59,872		
Allium*	57	0.24	6	EL	155	−15,288	30,886	− 217	973,215
				EUL	660	−14,891	31,102		
Primula*	185	0.43	6	EL	421	−43,494	87,831	− 801	466
				EUL	2256	−42,060	88,631		
Ficus*	112	0.36	5	EL	264	−14,185	28,897	− 963	851,445
				EUL	1140	−13,790	29,860		
Syzygium*	106	0.35	5	EL	254	−14,557	29,622	− 1085	45
				EUL	1090	−14,264	30,707		
Solanum*	187	0.31	7	EL	434	−39,049	78,967	− 1410	211.9 million
				EUL	2660	−37,528	80,376		
Caryophyllaceae*	225	0.29	7	EL	498	−67,977	136,951	− 1619	718.3 billion
				EUL	3108	−66,177	138,570		
Asplenium*	133	0.21	6	EL	317	−29,688	60,010	− 1758	8269
				EUL	1632	−29,252	61,768		
Eucalyptus*	136	0.23	6	EL	309	−14,683	29,984	− 1832	27
				EUL	1584	−14,324	31,816		
Ranunculus*	170	0.31	7	EL	400	−32,738	66,276	− 1986	1
				EUL	2422	−31,709	68,262		
Rhododendron*	117	0.35	7	EL	294	−22,049	44,687	− 2025	1034
				EUL	1680	−21,676	46,712		
Rosaceae*	529	0.30	7	EL	1118	−83,932	170,101	− 6350	1.72E + 23
				EUL	7448	−80,777	176,451		
Lichenized fungi	1317	0.44	9	EL	2712	− 499,157	1,003,737	− 8260	11,655
				EUL	23,760	− 482,238	1,011,997		
Primates	372	0.37	79	EL	1452	−648,012	1,298,927	−41,745	70.8 million
				EUL	59,250	−611,086	1,340,672		
Bats	815	0.15	29	EL	1888	− 589,602	1,182,979	−51,491	1.43E + 41
				EUL	47,444	−569,791	1,234,470		
Saxifragales	946	0.06	51	EL	2348	− 277,272	559,240	− 171,677	**
				EUL	96,798	−268,660	730,917		
Birds	7000	0.12	32	EL	14,285	−4,181,534	8,391,639	− 704,755	1.3E + 388
				EUL	448,192	−4,100,005	9,096,394		

#: column displays ΔAIC if ΔAIC favors the EUL model, and -ΔAIC otherwise. ^ and ^^: the number of loci differs from that reported in Table 1, because we discarded loci that contained fewer than the full complement of amino acids. ^: 7 loci were discarded; ^^: 2 loci were discarded. * and **: see Table 1. For methods used to compare the models, see text

## Discussion

The results of this analysis show that the phylogenetic trees inferred from empirical data sets often are found on large terraces of equally optimal trees, given certain assumptions about inference. The size of these terraces correlates inversely with data availability characteristics of the data: taxon sampling density, and a gene sampling “sufficiency” metric derived from theory. Evaluated by the latter measure, which incorporates the assumptions that no additional taxon sampling occurs and that sampling density does not increase, data sets seldom include enough genes to reduce terrace size to a single tree. We found that bootstrap support is reduced when the trees on terraces are included in replicate samples, and we showed how the distribution of taxon samples influences the size of terraces among data sets of otherwise similar sampling properties.

Our findings illustrate the frequency and scale at which terraces could arise from data sets assembled under a range of strategies. Of five high-coverage-density, genome- or transcriptome-scale data sets in our study sample (i.e., [34, 47, 48]) none induced terraces having more than one tree. Among low-density data sets extracted from the vascular plant megamatrix, terrace sizes varied widely; results from our taxon triple experiment show that distributions of samples across data partitions explain some of these differences. The findings for this group indicate the scale at which sets of equally optimal subtree topologies might emerge within the lower taxonomic ranks of trees inferred from extremely large (“mega”-scale) data sets. Our sample also included studies conducted at the ordinal or infra-class level (e.g., [33, 39, 41, 43]), some including large species samples to provide statistical power for downstream comparative analyses. Studies in this category rely on gene-rich, low-density data gathered predominantly from public repositories, but the gene samples, though large, do not achieve the depth required to reduce terrace size to one tree.

Tests [58] of the RAxML rapid and standard bootstrap search algorithms using empirical data have shown that, although the differences are small, rapid (heuristic) bootstrap search typically returns higher support values than standard bootstrap search; and when compared for the same data set, the total number of distinct bi-partitions in standard bootstrap samples is higher than that found in rapid bootstrap samples. The likely cause of these somewhat surprising differences in support levels is that the use of non-independent starting trees in rapid search “localizes” search in tree space and leads to stronger support [58]. These findings imply that, in tree space with terraces, standard bootstrap would overstate support less than does rapid bootstrap (i.e., values of *ρ* measured for basic and “terrace-aware” consensus trees would be nearer to one another). The effectiveness of search algorithm alternatives at estimating support in terraced tree space deserves further study.

A common approach to assembling data for the reconstruction of large and species-rich clades is to combine two data matrices of different coverage densities: a completely sampled matrix of many slow-evolving genes for a small set of taxa sharing most recent ancestors at deep nodes, and a sparser matrix of fast-evolving genes sampled for a larger collection of species concentrated in lower subclades (i.e., “top-down, bottom-up” sampling method [14, 34, 43, 46, 59]). Experiments using empirical data [14] show that this data sampling facilitates the accurate reconstruction of large clades at deep and shallow levels. Our taxon triple results suggest that terraces might arise from this sampling design if inclusion of the sparse complement - the fast-evolving genes - increases the number of taxon triples (relative to the base matrix) faster than the number of triples sampled within partition subsets. Similarly, combining incomplete taxa (taxa sampled for less than the full complement of genes) with a densely-sampled matrix of slow-evolving loci is thought to be advantageous for reconstructing deep nodes, since the incomplete data can subdivide long, saturated branches [6, 60, 61]. With this design, if blocks of the introduced taxon labels share few sampled genes - that is, if the incomplete taxa are sampled nonrandomly - we might expect terrace behaviors among the discontinuously sampled labels, as their inclusion would increase total data set taxon triples more than triples observed in partitions. Of course, in considering these common data assembly strategies, we leave aside other concerns arising from sparse or fragmented sampling (see Introduction).

Our results show that ΔAIC favored EUL models for some data sets, including many in which terrace analysis found terraces of a single tree; while favoring EL models for others, including many of lower sampling density, and most from which multi-tree terraces arose. The correlation between terraces in data sets and the information-based preference for simpler models deserves further study, but it is not unexpected, given the tendency of information-based model selection criteria to reject richer models where there are less data [56, 57].

### Inference models and “stands”

In this paper, we described the terraces that would arise in tree inference from real data sets under parsimony or ML-EUL inference assumptions. We also noted that one or more of the trees investigated were inferred originally with EL models, which do not induce terraces. There is an important connection between several concepts here that is independent of the particular inference model. We define a *stand* [27] as the collection of parent trees of a set of compatible input trees -- here the subtrees induced from the partitions. For trees inferred with parsimony or ML-EUL, all trees on a stand score the same, and all stands are terraces. For other inference models, the stands may include trees with different scores. Our study accurately characterizes the stands - their size, the variability of their member trees, their relation to decisiveness and other data properties - as they arise in a selection of empirical data sets. Stands occur in tree space because of the taxon coverage structure of a data set. Terraces form in stands because of the inference model (or inference method) decisions of analysts. We now consider briefly the matter of this inference model choice.

In practice, the default parameter settings of tree reconstruction programs may influence model use decisions. By default, RAxML [36] links edge-length parameters of partitioned models (defining an EL model), while unlinking all other parameters. In contrast, users of the maximum likelihood program IQ-Tree [62] must affirmatively choose among EL, PEL, and EUL classes of model. Users of PAML [63] can unlink all parameters, or may use any of a collection of linkage class combinations in which edge-length parameters are fixed to partial linkage [PEL]. Users of MrBayes [64] must actively unlink parameters, but can unlink all or any combination. Terraces can arise in inference with the latter two programs and can interact with program assumptions to affect outcomes: in PAML, the likelihood score calculated with an EUL model might belong to an entire collection of topologically distinct trees. Bayesian programs may infer higher posterior probabilities for some member trees of a terrace than others, for in the presence of missing data, Bayesian priors can favor the joining of long branches [27]. With PAML and MrBayes, it is unclear whether the mechanics of specifying model parameterization (described above) might incidentally “favor” some models more than others, but with RAxML, default settings may increase the frequency of EL model use. Our review of the inference methods used across our study sample suggests that if authors relied on inference program default settings, the rate of use of EL models was quite high.

#### Are EL inference models better for partitioned phylogenetic analyses?

The AIC model selection criterion favored EL models in several low-coverage data sets. Moreover, terraces have only been proven to arise under EUL models. Nonetheless, there are several reasons to think EL models are not guaranteed to provide better tree reconstruction outcomes, even for low-density data. One is that the results of EL inference are susceptible to multiple artifacts not related directly to terraces, such as those cited in the Background (e.g., affecting support, model parameter, and tree topology estimates). Second, in light of the prevalence of terraces, the “optimality” of a tree selected with EL from a poorly-differentiated likelihood surface (such as is likely to arise from low-density or low-information data) may be illusory, reflecting the imprecision of floating point arithmetic and stochasticity in the tree search process [36, 65]. For example, different addition orders of individual site log likelihoods can result in different summed log likelihood scores. Third, a number of studies have suggested that EL models may misspecify heterotachous evolutionary patterns. Broadly speaking, evolutionary biologists have defined heterotachy as within-site rate variation over time, but in phylogenetics, a substantial literature [66–72] has focused on heterotachous patterns in which intra-site variation can be observed as differences in branch-length patterns across loci. This “among-gene heterotachy” [73] naturally suggests the use of an inference model (a “heterotachy model” [27]) that separately parameterizes branch lengths for each data partition. Authors who have addressed the inferential problems posed by this form of heterotachy have often assumed that the identity of sites varying in common are not known in advance and must be inferred, and accordingly have used computationally intensive mixture models to sort sites into branch-length classifications (sets) [66–68, 70]. Models in this category, and others that optimize branch-length sets separately across partitions, have been found to recover better trees or to fit empirical or simulated data better than homogeneous (homotachous) models parameterized with single sets of branch lengths [18, 66–68, 70, 72, 73]. Several studies show that homogeneous models can become inconsistent under strong forms of between-locus heterotachy [68, 71, 74], and these findings have partly motivated efforts to formulate heterotachous models. Notably, some experimenters have inferred non-independence between genes and branch-length sets selected optimally with mixtures [73], and others have found that separate analyses conducted on broadly-defined functional categories of sequences exhibit substantially different branch-length patterns [72]. EUL parameterization seems a natural fit for these observed patterns. We urge further empirical studies addressing whether, generally, branch lengths covary among data partitions (i.e., rate shifts occur homogeneously across partitions, as would correspond with an EL model), or whether no such covariance exists, and within-partition branch length patters may be specified better with EUL parameterization. Some of the findings depended on complete data samples, but Sanderson et al. [27], using simulations to examine the effects of missing data, showed that while an edge-linked (EL) inference model correctly inferred trees from homotachous and strongly heterotachous data under full taxon coverage, the heterotachous (but not homotachous) data pattern misled the model when data were removed to form a pattern of partial taxon coverage. These results are consistent with studies undertaken in a variety of contexts showing that phylogenetic accuracy suffers from reliance on overly simple models [6, 74–78], and that missing data often worsens the effects of model misspecification [5, 6, 8, 27], at times misleading models that otherwise remain robust to violations of their assumptions. It is also the case that complex models (e.g., EUL models) may overfit the data [79–81], and this consideration motivated us to identify the edge-length model favored by the AIC model selection criterion for each data set in the study sample. The results, wherein the EUL model was preferred for higher-density data, are consistent with the expectation that richer data sets should support more complex models, but may also be indicative of differences in underlying evolutionary processes. For example, the preference for the EL model for most of the vascular plant submatrices may stem from using many of the same loci between data subsets. Similarly, evolutionary rate heterogeneity among the sampled loci may account for the choice of EUL models for genomic and transcriptomic data sets. Further study of model suitability and terrace formation may shed light on the relationship of terraces per se to the phenomenon of increased estimator variance or non-identifiability (sensu Rannala [82]) that can occur in inference with highly parameterized models. When terraces do arise, the ambiguity that they introduce into tree reconstruction can be mitigated by adding data, or can be integrated over to provide hypotheses for downstream evolutionary analysis.

### Remediating, summarizing, and analyzing terraces

#### Reducing terrace size

Given a tree *T* on a labels set *X*, and a set of subtrees *T|Y*_1_,…,*T|Y*_k_ induced by a taxon coverage pattern *Y*_1_,…,*Y*_k_, an algorithm adapted from the supertree literature can identify the smallest set of taxon labels to remove from X so that the subtrees T*|Y_1_,…,T*|Y_k_ define a reduced tree, T* [26]. Under the appropriate inference model, this stand of size one will then be a terrace of size one. This problem, of finding the maximum defining label set (MDLS), has an exact and easily computed solution for two induced subtrees (i.e., two loci). For data sets of more than two loci, applying the algorithm successively to pairs of loci gives an approximate solution [26, 27]. Taxa outside the MDLS, or outside the intersection of pairwise MDLSs, can be sampled for all loci, or their data removed. The MDLS solutions of trees in a confidence set, however, may differ from one another and from that of the original tree. Moreover, data augmented with new samples may imply a phylogeny not defined by its induced subtrees.

#### Fully sampling one gene

As noted earlier, a taxon coverage pattern is always decisive if any one gene is sampled for all taxa. However, full taxon coverage for one gene does not guarantee that the likelihood (or other score) surface will be well behaved; as noted in [26], a terrace-like flatness might characterize scores inferred from decisive data, if decisiveness is achieved with low-signal data restricted to a single gene.

#### Partitioning to reduce the size of terraces

Sanderson et al. [27] showed that for every data set, a unique maximal partition exists and that it corresponds to a maximal terrace (the largest terrace). Parsimony analyses should report the maximal partition and terrace, since parsimony scores would be unaffected. With maximum likelihood, partitioning to maximize model fit or performance may sometimes reduce the size of terraces. A procedure developed by Li et al. [79] for finding the optimal number of parameters in an inference model can reduce the number of partitions. With incompletely sampled data, reducing partition number should increase taxon coverage density and could reduce terrace size. In an analysis of genome-scale data, Xi et al. [59] used Bayesian inference of model mixtures to group sites by substitution pattern. The resulting partitioning scheme improved likelihood (as measured by AIC) and reduced terrace size compared to partitioning a priori by gene and codon position.

#### Summarizing and analyzing terraces

In addition to functions for computing consensuses, three terraphy package tools support terrace analysis and reporting. As noted earlier, an annotating tool reports the number of equally good subtree resolutions per clade of the strict consensus tree of a terrace. This feature decomposes terrace-based ambiguity into its combinatoric elements for analysis, and helps to highlight unresolved regions of large trees. Another feature constructs and outputs random trees sampled from a given terrace, allowing investigation of the impact of terraces on downstream comparative analyses. A third feature reports whether two trees belong to the same terrace. In conjunction with data from the strict consensus tree and taxon coverage matrix, the latter functionality could be used to detect the signature of another hazard of terraces, the supported, spurious clade. An experiment conducted on simulated data [27] showed that when sampling overlap between two clades is minimal – i.e., the groups share few sampled loci - the clades may be found merged in a large proportion of trees on the terrace. If many bootstrap trees fall on the terrace, the spurious clade in turn would occur at high frequency in the bootstrap sample. The data used for the experiment comprised two partitions of sequences patterned to ensure that bootstrap trees achieved identical scores, and translating the results to a prediction applicable to, for example, larger collections of loci or more complex patterns of sampling discontinuity would require further experiments. If an artifact of this type arose in real data, however, it would predict a conjunction of outcomes at a node: strong support among bootstrap replicate trees that reside on the terrace, and in the terrace consensus tree, weak resolution of the interior branches of the clade compared to a binary tree.

## Conclusion

Provided certain inference assumptions apply, the phylogenetic trees recovered from many large empirical data sets belong to large terraces of equally optimal trees. The size of these terraces varies inversely with two sampling properties of the data: taxon coverage density and gene sampling “sufficiency,” a measure derived from data decisiveness theory. Evaluated on this scale, which treats taxon sampling density and taxon count as fixed, data sets seldom include enough genes to reduce terrace size to one tree. At a given sampling density, a measure of the distribution of samples among genes can often predict the impact of terraces. The terraces found in bootstrap replicates can reduce resampling support for phylogenetic hypotheses. The widespread adoption of NGS approaches to data assembly will reduce incompleteness in data sets, and also the prevalence of terraces. A new program, terraphy, provides terrace discovery, analysis, and reporting tools.

The methods used originally by authors to reconstruct the published trees were variable and included inference with EL (edge-linked) models. Models of this type do not induce terraces, but for all data sets and starting trees surveyed, our findings characterize the collections (stands) of trees, many very large, as they arise from the data sets. Under EL inference, these trees have different scores. Under ML-EUL or parsimony inference assumptions, these collections of trees would be terraces. We used the AIC model choice criterion to determine the most appropriate edge-length model (EL or EUL) for each data set, and found that EUL models were preferred for some, including high-coverage data sets which induce single-tree terraces; whereas EL models were favored for others. The correspondence between single-tree terraces and selection of the EUL model is in line with expectations, given that sparse data may be insufficient to support complex models and also give rise to terraces. These model choice results, and the relationship between terraces and model suitability, deserve further study. If EL models are used to analyze low-density or low-signal data sets, terrace analysis can reveal potential ambiguity in the inference, for the likelihood surface recovered under such a data-and-model combination is likely to be poorly differentiated. The present study reveals the scale and frequency at which terraces would arise from parsimony or edge-unlinked maximum likelihood analyses of large data sets, and allows us to add terraces to the list of challenges in phylogenetic analysis from sparsely sampled and large collections of data.

## Abbreviations

AIC: Akaike Information Criterion
EL: Edge-linked
EUL: Edge-unlinked
MCC: Maximum clade credibility
MDLS: Maximum defining label set
ML-EUL: Maximum likelihood inference using an EUL model
NGS: Next-generation sequencing
PEL: Partially edge-linked
